# Effect of acetazolamide and subsequent ventriculo-peritoneal shunting on clinical signs and ventricular volumes in dogs with internal hydrocephalus

**DOI:** 10.1186/s13028-015-0137-8

**Published:** 2015-09-04

**Authors:** Malgorzata Kolecka, Nele Ondreka, Andreas Moritz, Martin Kramer, Martin J. Schmidt

**Affiliations:** Department of Veterinary Clinical Sciences, Small Animal Clinic, Justus-Liebig-University, Frankfurter Straße 108, 35392 Giessen, Germany

**Keywords:** Hydrocephalus, Carbonic anhydrase inhibitor, Ventriculo-peritoneal shunting, Cerebrospinal fluid

## Abstract

**Background:**

Acetazolamide is recommended for the reduction of cerebrospinal fluid production in canine internal hydrocephalus. The efficacy of the drug in terms of alleviation of the clinical symptoms and the restoration of normal ventricular volume has not been documented. We hypothesize that acetazolamide inadequately improve clinical signs and has no effect on the ventricular volume. Six dogs with internal hydrocephalus underwent neurological examination and were examined by magnetic resonance imaging, on the day of the diagnosis, after treatment with acetazolamide directly before surgery, and 6 weeks after implantation of a vetriculo-peritoneal shunt due to lack of improvement after medical therapy with 10 mg/kg acetazolamide three times daily (TID). The ventricular volume in relation to the total brain volume was determined on each occasion. The changes in relative ventricular volume and of the neurological status were assessed and compared.

**Results:**

McNemar’s test revealed no significant differences in clinical symptoms before and after medical treatment (*P* > 0.05). However, clinical symptoms changed significantly after surgical treatment (*P* = 0.001). The ventricle-brain ratio was not significantly changed after therapy with acetazolamide (*P* > 0.05); however, after subsequent shunt implantation, it was significantly reduced (*P* = 0.001).

**Conclusion:**

Acetazolamide (10 mg/kg TID) showed no effects on clinical signs or ventricular volume in dogs with internal hydrocephalus. After subsequent ventriculo-peritoneal shunting, the dogs had a significantly reduced cerebral ventricular volume and five out of six dogs had no abnormal findings in neurological examination.

## Background

Internal hydrocephalus is the most common congenital anomaly of the central nervous system in dogs [1–5]. In idiopathic communicating hydrocephalus, a mismatch between production and absorption leads to the accumulation of cerebrospinal fluid (CSF), increase of intraventricular pressure, and dilation of the ventricular system in affected animals. The underlying causes for a decreased absorption rate of CSF have not been identified yet [1, 2]. Medical therapy for the lowering of high ventricular pressure which aims to reduce CSF production through the use of glucocorticoids and diuretics has been proposed as a reasonable treatment option in hydrocephalic dogs [2–4]. The carbonic anhydrase inhibitor acetazolamide also has been proposed for medical treatment by some authors [2–4].

To objectively assess the efficacy of acetazolamide, we compared the development of clinical signs and ventricular volume before and after treatment with acetazolamide and after ventriculo-peritoneal shunting.

## Methods

Between 2000 and 2013, 59 dogs were diagnosed with idiopathic communicating internal hydrocephalus at the Clinic for Small Animals of the Department of Veterinary Clinical Sciences, Justus-Liebig-University, Giessen, Germany. All dogs underwent magnetic resonance imaging (MRI) of the head after owners’ approval. The dogs were sedated with 0.1 ml/kg diazepam^1^ and 0.3 ml/kg propofol^2^ given intravenously. General anaesthesia was maintained after endotracheal intubation by inhalation of isoflurane in oxygen. MRI was performed using a 1.0 Tesla scanner^3^ and C3 coil. The diagnosis of idiopathic communicating hydrocephalus was based on severely dilated lateral ventricles in association with patency of the interventricular foramen and mesencephalic aqueduct. Cases were included as ‘severe hydrocephalus’ if signs of high pressure were present, e.g. periventricular oedema, dorsal dislocation of the corpus callosum, and narrowing of the sulci and gyri as reported [6].

Portosystemic shunt was routinely ruled out in all dogs using in house ammonia and bile acid testing. CSF examination was within normal limits (<5 cells/µl, red blood count <1500/µl, protein <300 mg/l, specific gravity 1004–1012, and no cytological abnormalities). From transverse, dorsal and sagittal T2-weighted images (T2-Turbospin echo, TE 120 ms, TR: 2900 ms, slice thickness 2.5 mm, field of view 160 × 160 mm, matrix 288 × 288), transverse FLAIR and transverse T1-3D fast field echo (FFE) sequences (TR 24.24, TE 6.9 ms, field of view 160 × 160, matrix 672 × 672, slice thickness 1 mm) FFE images of the head were chosen for image segmentation and volume determination. Image processing for volume rendering was achieved using graphical software^4^. This program allows accurate manual image segmentation on a slice-by-slice basis. All voxels corresponding to a single anatomical structure in the images are selected and assigned to the same value in the mask. The final mask thus contains information about all the selected anatomical structures in combination with the original data. Polygonal surface reconstruction algorithms allow the determination of the volumes of different structures in the images. MRI-based volume measurements of the various brain structures are routinely performed in veterinary medicine. The accuracy of the technique even for small volumes has been proven in veterinary neuroradiology [7, 8].

**Fig. 1:**
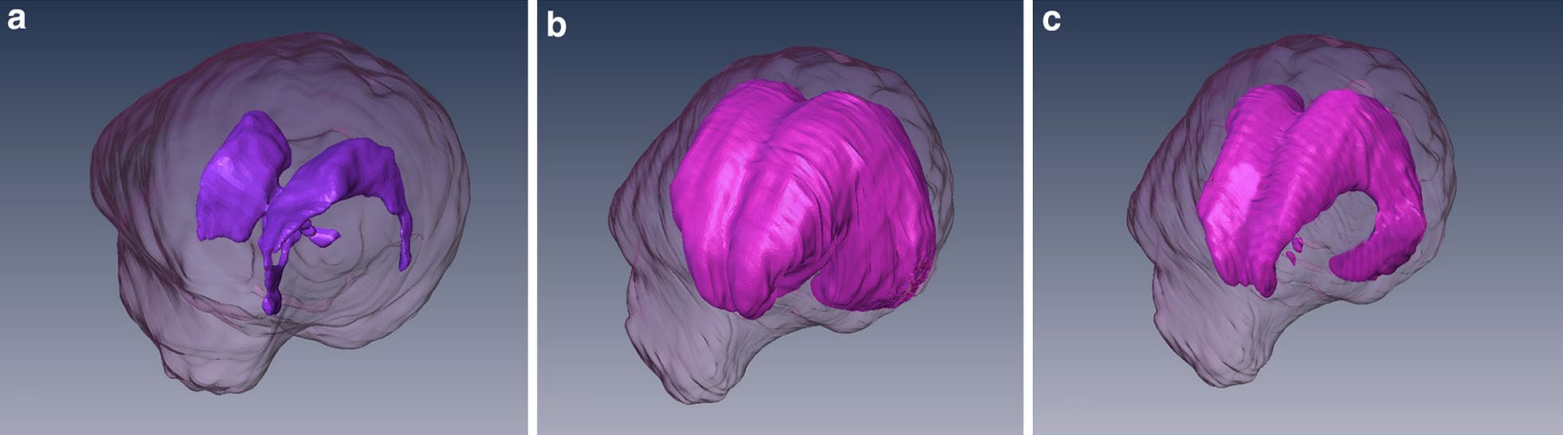
3D models of the brain and ventricular system of dogs based on manual segmentation of the two dimensional outlines of the ventricular system in MR-images: **a** Cavalier King Charles spaniel with normal ventricular dimensions. **b** Boston terrier with internal hydrocephalus before and after (**c**) implantation of a ventriculo-peritoneal shunt (VPS). The cerebral parenchyma is transparent

The total ventricular volume was calculated and expressed in relation to the volume of the brain including cerebrum, cerebellum, and brainstem (ventricle-brain ratio). The total ventricular volume comprised all ventricles including the choroid plexus. The delineation of both the ventricular system and the brain parenchyma to the spinal cord was set along a vertical line at the obex. The segmented brain and ventricle partitions are written and graphically presented by the program (Fig. 1).

After discussion of treatment options, six owners decided for medical management because of concerns of potential operation risk. The dogs were treated with 10 mg/kg acetazolamide^5^ three times daily (TID). A thorough clinical control of the dogs’ clinical status and reassessment of the ventricular dimensions using MRI was recommended. Owners agreed to present the dog for a re-examination after 6 weeks of medical treatment. Due to a lack of improvement of clinical signs at reexamination, owners requested surgical therapy using a ventriculo-peritoneal shunt system.^6^ MRI was repeated before surgery to reassess the status of the ventricular dilatation, for surgical planning, and for later assessment of ventricular reconstitution.

For the ventriculo-peritoneal shunt placing every patient was placed in the right lateral recumbency. The fur on the head, cervical region, thorax and abdomen was removed and the skin was disinfected. After rostrotentorial approach a 5 mm hole was performed with a pneumatic drill in the skull at the level of the ectosylvian or suprasylvian gyrus after careful determination of the implantation site in MRI. A cut with a scalpel blade was performed to open the dura and haemorrhage was eliminated with a bipolar cautery. Commercially available ventricular catheter with a stylet was placed into the left ventricle through the cerebral cortex. The depth and the angle of the insertion were determined prior to surgery in MRI. Another end of the catheter was tunnelled beneath the masticatory muscle and subcutaneous to lateral cervical area and connected to the CSF port that enables transcutaneous CSF collection and further to the peritoneal catheter. Peritoneal catheter was placed in the peritoneal cavity caudal to the last rib and attached with a Chinese finger-trap suture.

The medical history and the degree of neurological signs before and after medical and surgical treatment were recorded using standardised examination protocols. The clinical examination was performed by a board-certified neurologist following a standardised protocol. Clinical and MRI reexamination of the brain was performed 6 weeks after surgery. The ventricle-brain ratio was again determined, based on the postoperative MRI.

Statistical analysis was performed using a commercial software package^7^. As the statistical distributions of the measured values were skewed to the right and the data did not show homogeneity of variance, a logarithmic transformation was performed at the beginning of the analysis. Following a one-factorial analysis of variance with repeated measures to test for global differences, the treatments were compared pairwise using the Student–Newman–Keul’s test. Two way frequency tables were created and analysed with a one-sided exact McNemar test to compare clinical signs (present or not present) before and after surgical and medical treatment. *P* < 0.05 was considered statistically significant.

## Results

Age, breed, gender, and clinical signs before and after medical and surgical therapy are summarised in Table 1. McNemar’s test revealed no significant differences in clinical signs before and after medical treatment (*P* > 0.05). However, there was significant improvement of the clinical signs after surgical treatment (*P* = 0.001). The ventricle-brain ratio was not significantly changed after therapy with acetazolamide (*P* > 0.05); however, after subsequent shunt implantation, it was significantly reduced (*P* = 0.001).

**Table 1:** Summary of the epidemiological data, clinical signs and ventricle-brain ration before and after acetazolamide therapy and after surgical therapy in six dogs with idiopathic internal hydrocephalus

Breed	Gender	Age (weeks)	Neurologic signs on admission	Duration of signs (weeks)	Neurologic signs 6 weeks after acetazolamide therapy	Neurologic signs 6 weeks after surgery	Ventricle-brain ratio before treatment	Ventricle-brain ratio after acetozaolamide therapy	Ventricle-brain ratio after surgery
Chihuahua	Male intact	12	Obtundation circling, bilateral ventrolateral strabismus	~8	Obtundation circling, bilateral ventrolateral strabismus	None	0.26	0.3	0.05
Boston Terrier	Male intact	22	Obtundation, circling, aimless barking	~4	Obtundation, circling, aimless barking	None	0.5	0.48	0.5
Jack Russel Terrier	Female intact	18	Posttectal visual deficits, absent menace response	~10	Posttectal visual deficits, absent menace response	Posttectal visual deficits, absent menace response	0.3	0.35	0.08
Chihuahua	Female intact	21	Obtundation, mild ataxia on all four limbs	~12	Obtundation, mild ataxia on all four limbs	None	0.35	0.4	0.04
Mixed breed	Female intact	20	Obtundation, mild ataxia on all four limbs, hypermetric gait	~8	Obtundation, mild ataxia on all four limbs, hypermetric gait	None	0.33	0.36	0.03
Dachshund	Female intact	28	Visual deficits, reduced menace response	~12	Visual deficits, reduced menace response	None	0.36	0.4	0.08

## Discussion

Medical and surgical treatment options exist for the reduction of intraventricular CSF pressure in dogs with idiopathic internal hydrocephalus [1–4]. Treatment failure with acetazolamide was reported for human infants and incidentally for a dog [9, 10]. As distinct information about treatment success of acetazolamide therapy is lacking, we prospectively assessed the efficacy of medical treatment in comparison with subsequent ventriculo-peritoneal shunting. We found that the ventricular volume and the clinical signs of dogs with internal hydrocephalus were not sufficiently changed using acetazolamide 10 mg/kg TID. In contrast, the subsequent implantation of a ventriculo-peritoneal shunt reduced both ventricular volume and clinical signs in the same dogs with exception of the Boston terrier, in which clinical signs improved, but the ventricle-brain ratio has not changed.

It has already been shown that the use of acetazolamide to reduce or improve clinical signs is ineffective in a large group of human infants with congenital hydrocephalus [9]. Progression of clinical signs in dogs with hydrocephalus treated with acetazolamide was also incidentally reported [10]. It has been experimentally shown that the concentration of carbonic anhydrase in the choroid plexus of cats must be lowered by at least 99.95 % of its full activity to reduce CSF production significantly [13]. Having a half-life of 2 h and duration of action of 6–8 h [14], the full effect of carbonic anhydrase inhibition may not be sufficiently long-lived in the dog to constantly reduce CSF production. Higher doses and administration every 6 h might be more successful. Although the reduction of CSF production has been shown in dogs, none of these experimental studies measured the production for longer than 12 h [15, 16]. If the period of observation had been longer, CSF production might have returned to higher levels in these dogs, too.

Adaptive processes such as increased production of osmogenic ions from the ependyma and choroid plexus or the upregulation of an acetazolamide-resistant isozyme, which could be demonstrated in the epithelial cells of the rodent and human choroid plexuses [17], might also be an explanation for the lack of treatment success.

For the cases presented herein, the postoperative changes in ventricular volume can be attributed to the adjustments of the tissue to the reduced intraventricular pressure condition. In addition to the clinical course of the patient, reexpansion of the brain parenchyma and reduction of the ventricular volume serve as important morphological indicators for success or failure of CSF shunting surgery in human medicine [11]. Our findings match those reported from shunting procedures in children that showed significantly decreased ventricular volumes as early as the first 3 weeks after shunt implantation [11]. In case of Boston terrier the reason for the missing reduction is unclear. Reduction of pressure must not automatically lead to a restauration of brain parenchyma that might already have undergone atrophy under pressure. Reduction of oedema and mild changes of ventricular volume might have been enough for improvement of unspecific neurological clinical signs like aimless barking or obtundation.

After induction of experimental hydrocephalus and ventriculo-peritoneal shunting in dogs, recovery of ventricular size and gross configuration of the brain was observed mainly as a result of an increased volume of white matter due to regeneration of myelin sheaths [12]. Remyelination did not occur in chronic hydrocephalus after widespread destruction of axons. Hence, early shunting at the age of 2–3 months was considered to be of the utmost importance in the prevention of permanent tissue damage [12]. The definite time point at which reduction of high intraventricular pressure no longer leads to tissue reconstitution has not been determined yet. However, irreversible damage to the axon of the white matter at the time of or during acetazolamide therapy is unlikely in our patients, as subsequent shunt implantation could successfully reduce ventricular volume in all and improve clinical signs in five out of six dogs. Although ventricular volume decreased in one dog, its visual impairment and absent menace response persisted. Visual deficits and blindness have been reported to respond poorly to ventriculo-peritoneal shunting in dogs and cats [5]. Processing and recognition of visual information is complex and requires full functional integrity of several cortical fields and their white matter connections. Irreversible damage to one or more visual structures including the optic radiation, primary and secondary visual cortex, and white matter pathways that connect visual cortical areas might be responsible for permanent visual deficits in this dog. This damage can remain after intraventricular pressure reduction and reconstitution of other parts of cerebral white matter.

The relatively low number of the patients in the concurrent study has to be considered as a major limiting factor. Medical treatment was performed with acetozolamide with the common dose of 10 mg/kg because this dose is recommended in text books. We may only have shown that a dose of 10 mg/kg is ineffective for treatment of internal hydrocephalus. Other doses were not tested. In an optimum manner two groups with two different doses of acetazolamide should have been tested. To assess the efficacy of the medical treatment the measurement of the CSF production would be optimal but unfortunately it is only possible in experimental studies. It is possible that CSF reduction after medical therapy occurs but not in a sufficient amount to have an influence on the reduction of the ventricle size.

## Conclusion

Based on the results of this study we conclude that medical treatment with 10 mg/kg TID acetazolamide cannot sufficiently abolish neurological signs or reduce ventricular size in dogs with idiopathic communicating internal hydrocephalus. In contrast to this subsequent shunt implantation reduced ventricular size and improved clinical symptoms.

## Abbreviations

CSF: cerebrospinal fluid
FFE: fast field echo
FLAIR: fluid-attenuated inversion recovery
MRI: magnetic resonance imaging
TID: three times daily
TE: time of echo
TR: time of repetition

## Footnotes

1 Diazepam rationpharm, Ratiopharm GmbH, Ulm , Germany.

2 Propoflo Ahlbrecht Albrecht GmbH, Aulendorf, Germany.

3 Gyroscan Intera, Philips Medical Systems, Hamburg, Germany.

4 AMIRA®, Visual Concepts, Berlin, Germany.

5 Diamox, Haupt Pharma, Wolfratshausen, Germany.

6 paediGav, Christoph Miethke GmbH und Co. KG, Potsdam, Germany.

7 Graph Pad Prism 4.0, Graph Pad Software Inc., San Diego, California.
